# Immunogenicity of induced pluripotent stem cell-derived smooth muscle cells results from a reduction in the expression of indoleamine 2,3 dioxygenase (IDO-1)

**DOI:** 10.1080/17460751.2026.2631599

**Published:** 2026-02-25

**Authors:** Katherine Partridge, Vera J. Mehler, Chris J. Burns, Jordan S. Pober, Melanie L. Moore

**Affiliations:** a Science and Research, Medicines and Healthcare Products Regulatory Agency (MHRA), South Mimms, UK; b Department of Immunobiology, Yale School of Medicine, New Haven, CT, USA

**Keywords:** Induced pluripotent stem cells (iPSC), iPSC, IDO-1, smooth muscle cells, immunology, characterization

## Abstract

**Background:**

Smooth muscle cells (SMCs) derived from allogeneic induced pluripotent stem cells (iPSC) hold significant potential in cellular therapy for many cardiovascular diseases. However, their immunological profile is poorly characterized, limiting their clinical progression.

**Aims:**

This study aimed to investigate the immunogenicity of iPSC-SMC in comparison with naturally derived vascular SMCs (v-SMCs).

**Result:**

We found iPSC-SMCs, in contrast to naturally derived vascular SMCs (v-SMC), triggered T effector memory (T_EM_) cell proliferation. However, expression of T_EM_ activation-related proteins was comparable between both cell types. Since arterial v-SMCs can also establish immunoprivilege through indoleamine 2,3 dioxygenase (IDO-1) activity, we therefore investigated IDO-1 expression in two independently engineered iPSC-SMCs (NIBSC 8 (N8) and Yale 6 (Y6)). IDO-1 expression and functionality was markedly reduced in both iPSC-SMC lines compared to v-SMC and unlike v-SMC, neither iPSC-SMC line could modulate the immune response in a co-culture with CD3/CD28 activated peripheral blood mononuclear cells (PBMC).

**Conclusions:**

These results indicate that iPSC-SMC’s impaired ability to modulate the immune response through IDO-1 expression contributes to their differing immunogenicity to v-SMC and highlights the importance of immune phenotyping for therapeutic applications of iPSC-derivatives.

## 1. Introduction

Smooth muscle cells (SMCs) are a vital component of large blood vessels. As a key part of the vascular system, SMC dysfunction is associated with many cardiovascular disease states including atherosclerosis, focal stenoses, aneurysm, hypertension, and shock [1–6]. Despite extensive research into treatment strategies, the most common treatments for localized dysfunction of a vascular segment such as a coronary artery consist of stents, angioplasties, and bypass vein grafts [3,7]. None of these treatments fully replicates a normal functioning arterial segment. In more recent years, the development of tissue engineered vascular grafts (TEVGs) has sought to overcome many of these complications [3–5,8]. However, these too are constrained by challenges, including the development of physiologically and immunologically suitable 3D structures, and the sourcing of functional cells in large enough numbers for therapeutic use [3].

Pluripotent stem cells (PSCs), such as embryonic stem cells (ESCs) and induced pluripotent stem cells (iPSCs), have been used extensively in disease modeling and drug discovery [7,9–11] and are promising candidates for the next generation of tissue engineering approaches for the treatment of many of conditions listed above. iPSCs have been of particular interest since their first development in 2006 [12], as they are derived from adult somatic cells, circumventing the ethical concerns raised using ESCs and can enable personalized medicine strategies.

Before the wide scale therapeutic application of cells derived from iPSCs, certain key aspects of these cells will need to be characterized. This is primarily because the concept of “cell reprogramming” remains relatively poorly understood. In addition to functionality after differentiation, the propensity to trigger an immune reaction in the host is of great importance [13]. Vascular SMCs (v-SMC) are innately immunomodulatory, due to a lack of co-stimulatory molecule expression and interferon-gamma (IFN-γ)-inducible expression of indoleamine 2,3 dioxygenase (IDO-1) [14–16]. In contrast, data suggest that iPSC-SMCs are immunogenic [17–20]. In our previous investigations to determine the immunogenicity of iPSC-derived neural crest stem cells [21], which showed negligible immunogenicity, smooth muscle cells, derived from the same iPSC lines, caused immune cell activation in the same assays, supporting previously published data [17,20]

Here, we investigate the underlying mechanisms behind the immunogenicity of iPSC-SMCs. Our data suggest that a key difference between iPSC-SMCs and v-SMCs is the failure of the former to express IDO.

## 2. Experimental procedures (methods)

### 2.1. Cell culture – SMC and endothelial cells

NIBSC 8 (N8) iPSC-SMCs were differentiated from N8 iPSCs [6] (donated by the UK Stem Cell Bank, Potters Bar, UK; Certificate of Analysis is provided in Supplemental Information) using a monolayer method and characterized as previously described [21–23]. Briefly, iPSCs were cultured to confluency in mTeSR medium on Matrigel-coated plates. Cells were then cultured with CHIR99021 and BMP-4 to initiate differentiation (Day 0). From Day 3–7 cells were cultured with VEGF-A and FBF-β in RPMI1640 supplemented with 2% B27 – insulin, and from Day 7–14, with PDGF-β and TGF-β in supplemented RPMI1640 medium. Cells were then purified for iPSC-SMCs by maintaining them in 4 mM Lactate RPMI1640 for 4–6 days [22]. iPSC-SMCs were then characterized through immunofluorescence staining for the key smooth muscle cell marker, smooth muscle actin (SMA), as well as qPCR for *SMA* and additional SMC markers *CALPONIN* and *TRANSGELIN* and the endothelial marker *VE CADHERIN* (data previously published in Supplemental Information of Mehler et al., [21]). Vessel-derived SMCs, SMCs differentiated from the Y6 iPSC line were generously donated by Professor George Tellides and Dr Yibing Qyang respectively (Yale University School of Medicine). Yale 6 (Y6) iPSC-derived SMCs (designated Y6 iPSC-SMC) were generated from iPSCs using an optimized embryoid body (EB) method and characterized as previously described by Dash, et al., [24,25]. Briefly, from iPSCs, grown in feeder-free culture, EBs were prepared in a mixture of mTESR1 and EB differentiation medium (DMEM +10% FBS +1% non-essential amino acids (v/v) + 2 mM L-glutamine and 0.012 mM 2-mercaptoethanol) at a ratio of 1:3. After 2 days culture, prepared EBs were then cultured in EB differentiation media for 3 days before transferring to gelatin coated plates and culturing for 5 days. EBs were then cultured for a further 7 days in SmGM-2 medium (Lonza) [24]. Resulting iPSC-SMCs were characterized with immunofluorescence staining for calponin, SMA, transgelin (SM22α), elastin, and smooth muscle myosin heavy chain (MHC), and by fluorescence activated cell sorting (FACS) for calponin and SMA (data previously published in Supplemental information of Dash et al.,). Human umbilical vein endothelial cells (HUVECs) were purchased from the Yale vascular cell culture core. Further details of cell maintenance and passage are provided in the Supplemental Information.

### 2.2. Cell culture – BMMSC

Bone marrow derived mesenchymal stromal cells (BMMSCs) were derived from bone marrow aspirates (AllCells, Alameda, CA, USA) and characterized as previously described [21,26]. Further details of cell maintenance and passage are provided in the Supplemental Information.

### 2.3. Peripheral blood mononuclear cells (PBMC)

Donor blood and isolated peripheral blood mononuclear cells (PBMCs) were handled in accordance with the Human Tissue Act (2004) and with project approval from the internal Human Materials Advisory Committee at the National Institute for Biological Standards and Control. PBMC were isolated using Histopaque®-1077 (Sigma-Aldrich) from whole blood from in-house donors (NIBSC), leucocyte cones from the NHS Blood and Transfusion Service (NHSBT, UK), or anonymized adult volunteer donors collected by the Yale New Haven Medical Centre Blood Bank (USA).

### 2.4. Immunophenotyping and IDO-1 flow cytometry

Cells were harvested using TrypLE™ Express Enzyme (1X) and washed in FACS wash buffer (phosphate buffered saline (PBS), 2% FCS, 2 mM EDTA). Immune related antigen expression was analyzed by flow cytometry, staining for surface markers; HLA-ABC, CD86 (BD Biosciences, Berkshire, UK), LFA-3 (Abcam), OX40-L (R&D systems) or ICOS-L (eBioscience) [27]. Cells were then fixed with 4% paraformaldehyde (PFA) in PBS and analyzed using BD FACS Canto II (BD Biosciences). Data were analyzed using FlowJo 7.6.5.

To assess the expression of IDO-1, v-SMCs, HUVECs, N8 iPSC-SMCs and Y6 iPSC-SMCs were split to 6-well plates at a density of 3-5x10^5^ cells/ml and 50 ng/mL IFN-γ was added to test wells or untreated as a control. Cells were incubated for 72hrs at 37°C in 5% CO_2_ before collection by trypsinization and staining with the fixable viability dye (FVD, eFluor 660, Invitrogen) followed by intracellular staining for IDO-1 or an isotype control (ThermoFisher). Samples were analyzed using BD FACS Canto II. Data was analyzed using FlowJo 10.8.1.

### 2.5. T cell proliferation and immunosuppression assays

Immunogenicity of N8 iPSC-SMCs was assessed via T cell proliferation assays in which whole PBMC or isolated T cells were used as responder cells, with SMCs serving as stimulator cells and autologous/allogeneic PBMCs used as negative and positive control, respectively. Cells were co-cultured for 5 or 7 days, with or without inclusion of 6 µM the IDO-1 inhibitor NLG919 (Navoximod, AbCam, UK) and further details are provided in Supplemental Information. PBMC harvested at the end of co-culture were stained with the fixable viability dye, eFluor660 (FVD, eFluor 660, Invitrogen), and the surface T cell markers CD3, CD4, CD8, and CCR7 (BD Biosciences) and analyzed using BD FACS Canto II. Data were analyzed using FlowJo 7.6.5.

Immune-suppression was assessed in the same assays, but with additional activation stimulus provided by the inclusion of Dynabeads^Ⓡ^ Human T-Activator CD3/CD28 (Gibco™) at the start of co-culture. Further details are provided in the Supplemental Information.

### 2.6. Quantitative polymerase chain reaction (qPCR)

Quantitative polymerase chain reaction (qPCR) for IDO expression was performed as previously described [21] on the Rotor-Gene Q Thermocycler, and data analyzed using Rotor-Gene Q System (QIAGEN, London, UK), 2.1.0. Further details are provided in the Supplemental Information

### 2.7. Reverse-phase high performance liquid chromatography (RP-HPLC)

Kynurenine and tryptophan content in supernatants from cell cultures was measured by HPLC as a readout of IDO-1 enzyme activity. Samples were prepared as described in the Supplementary Information. Filtered samples were loaded into a Thermo Scientific UltiMate 3000 System, with an Agilent Zorbax SB-C18 column, 4.6x150 mm, 3.5 µm (Agilent, Oxford, UK), controlled by, and analyzed on Chromeleon 7 Software. L-Trp and L-Kyn (Sigma-Aldrich) were diluted to form a standard curve for both products to enable peak identification. Further details are provided in the Supplemental Information

### 2.8. Statistical analysis

Values were expressed as mean±SEM (standard error of the mean). The significance of differences among multiple groups was determined with a one-way analysis of variance (ANOVA) or two-way ANOVA followed by a Bonferroni or Dunnett’s multiple comparisons posttest (GraphPad Prism, 8.2.1). Differences were considered significant where *p* < 0.05. Significance levels defined as follows: *= *p* ≤ 0.05, **= *p* ≤ 0.01, ***= *p* ≤ 0.001, ****= *p* ≤ 0.0001.

## 3. Results

### 3.1. Immune response to iPSC-SMC and their naturally differentiated counterpart, vascular SMC

To investigate the immunogenic properties of iPSC-SMCs, the proliferation of allogeneic T cells in response to iPSC-SMCs was measured (Supplementary Figure S1). Whole peripheral blood mononuclear cells (PBMCs) were used as the responder population, using NIBSC8 (N8) iPSC-SMCs as stimulator cells, with autologous and allogeneic PBMCs used as negative and positive stimulator cell controls, respectively. Proliferation was quantified by measuring levels of Kiel67+ (Ki-67^+^) CD3^+^ and CD3^+^CD8^+^ populations. Human Ki-67 protein expression is strictly associated with proliferation, thus Ki-67^+^ cells denote proliferating cells [28]. Responses were assessed as a Stimulation Index (SI), calculated as the number of Ki-67^+^ cells of each T cell population in the different co-cultures, divided by the number of Ki-67^+^ cells in the autologous control. An SI > 2 was considered positive. Active T cell proliferation was observed in response to N8 iPSC-SMCs (SI 2.57 ± 0.50) at a level similar to the allogeneic PBMC stimulator control (SI 2.16 ± 0.29). A greater level of proliferation was seen in the CD3^+^CD8^+^ T cell population, with N8 iPSC-SMCs giving an SI of 11.49 ± 4.8. These data are consistent with the findings of Mehler et al., [21].

**Figure 1.**
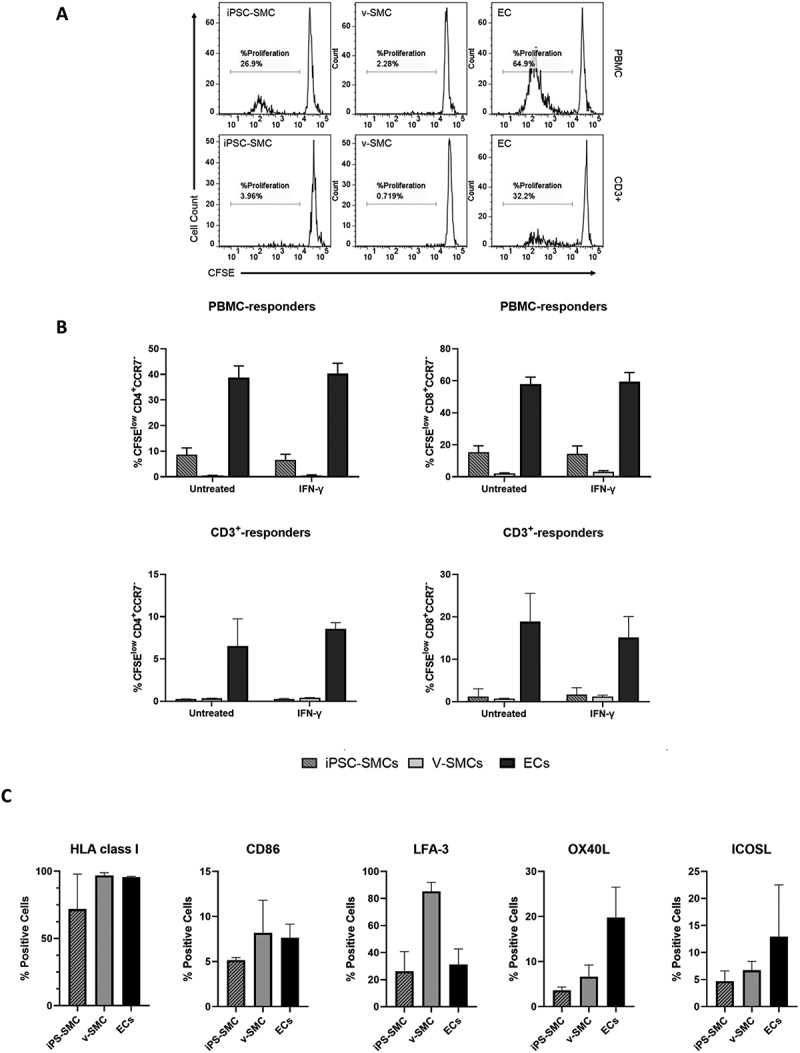
Comparison of immunogenic potential between induced pluripotent stem cell derived smooth muscle cells (iPSC-SMCs), vascular smooth muscle cells (v-SMCs) and endothelial cells (ECs) (A) Representative histograms show levels of CD8+ T_EM_ cell proliferation of isolated CD3+ T cells or peripheral blood mononuclear cells (PBMCs) as responder cells after 7 days co-culture with N8 iPSC-SMC, v-SMC or ECs as stimulator cells. %CFSE^low^ denoted proliferating cells. (B) Graphs show %CFSE^low^ cells of CD4+CCR7- cells and CD8+CCR7- cells to different stimulator cells. Error bars represent ±SEM (*n* = 4 from two independent experiments). ECs were used as a positive control and induced proliferation in all conditions. (C) Graphs showing percentage expression of immune related antigens HLA class I, CD86, LFA-3, OX40-L and ICOS-L in N8 iPSC-SMC, v-SMC and EC populations assessed via flow cytometry. Error bars represent ±SEM (*n* = 2 from independent experiments).

T effector memory cells (T_EM_) have been shown to play a major role in acute allograft rejection [29,30]. To further investigate the response of the T_EM_ population to iPSC-SMCs we co-cultured the iPSC-SMCs or v-SMCs with either whole PBMC or isolated CD3^+^ T cells, specifically analyzing the proliferation of T_EM_ cell populations (CD4^+^CCR7^−^ and CD8^+^CCR7^−^), using carboxyfluorescein succinimidyl ester (CFSE) dilution as readout of proliferation (Figure 1). In this case, endothelial cells (ECs), an immunogenic cell type, were used as a positive control [15,31]. SMC and EC populations were assessed untreated, or after treatment with IFN-γ. ECs consistently stimulated the highest level of T_EM_ cell proliferation (Figure 1(B)). When PBMCs were used as responder cells, iPSC-SMCs triggered proliferation of both CD4 T_EM_ (6.6–8.6%) and CD8 T_EM_ (14.3–15.4%) whereas v-SMCs induced negligible proliferation of CD4 T_EM_ (0.4–0.5%) and much lower proliferation of CD8 T_EM_ (2.0–3.0%) (Figure 1(B)). While ECs induced proliferation with isolated CD3^+^ as responder cells (6.5–8.6% of CD4 T_EM_ and 15.1–18.9% of CD8 T_EM_), iPSC-SMC and v-SMC triggered negligible proliferation regardless of whether SMC populations had been previously exposed to IFN-γ (0.24–0.42% CD4 T_EM_ cells and 0.7–1.6% CD8 T_EM_ cells).

To investigate the basis of these differences in immunogenicity, N8 iPSC-SMC were compared to v-SMC and ECs for the expression of a panel of proteins involved in T_EM_ cell activation including O×40 ligand (OX40-L), inducible costimulator ligand (ICOS-L), leukocyte function-associated antigen-3 (LFA-3), human leukocyte antigen-1 (HLA-I), and CD86. Data are presented in Figure 1(C) and with histograms from one representative assay presented in supplementary figure S2A, along with corrected mean fluorescence intensity (MFI) in figure S2B. HLA-I expression levels were broadly comparable between the cell types (mean expression of 71.8%, 96.8%, and 95.6% iPSC-SMC, v-SMC, and EC, respectively), as was CD86 expression (5.2% in iPSC-SMC; 8.2% in v-SMCs; and 7.6% in ECs), although notably, expression level of these markers (MFI) was higher for in v-SMC for both HLA Class I and CD86 (Fig S2B). LFA-3 showed a markedly higher expression level in v-SMCs (84–85%) compared to iPSC-SMCs (25–26%) or ECs (27–31%). OX40-L, on the other hand, was expressed by ECs (19.8%), but not to the same extent by iPSC-SMC and v-SMCs (6.6% and 3.6%, respectively). These same trends are echoed in the expression levels of these proteins (MFI) from the same experiments (Fig S2B). It is noted that OX40-L has previously been correlated with enhancing an allogeneic CD8 T_EM-_cell response to v-SMCs [16] and that this was only expressed at negligible levels by iPSC-SMCs. The percentage of ICOS-L^+^ cells showed a similar pattern, where lower expression was observed in iPSC-SMCs (4.7%) and v-SMCs (6.7%) and to a greater extent in ECs (12.9%) (Figure 1(C)); however, expression of ICOSL appeared higher for v-SMC (Fig S2B). Overall, iPSC-SMCs showed lower expression levels than either v-SMCs or ECs, suggesting that quantitative differences in expression levels cannot explain differences in immunogenicity.

### 3.2. Indoleamine 2,3-dioxygenase-1 (IDO-1) activity

The limited immunogenicity of v-SMCs and mesenchymal stromal cells (MSCs) has been shown to correlate with the expression IDO-1 [14,32]. In MSCs, IDO-1 expression is responsible for inhibiting T cell proliferation, Cytotoxic T Lymphocytes (CTL) formation, and promoting an anti-inflammatory phenotype through cytokines such as IL10 and TGF-β [33]. The ability of IDO-1 to inhibit T cell proliferation is linked to its activity in the depletion of tryptophan, an essential metabolite used by T cells [34–36]. Tryptophan catabolism is initiated by IDO-1 catalyzing the conversion of tryptophan to N-formyl kynurenine which can be subsequently deformylated to kynurenine [34]. In an experimental setting, we can infer immunosuppressive activity by looking at both IDO-1 expression in cells, as well as at the relative levels of tryptophan (Trp) and kynurenine (Kyn) in the supernatant of cell cultures [36].

**Figure 2.**
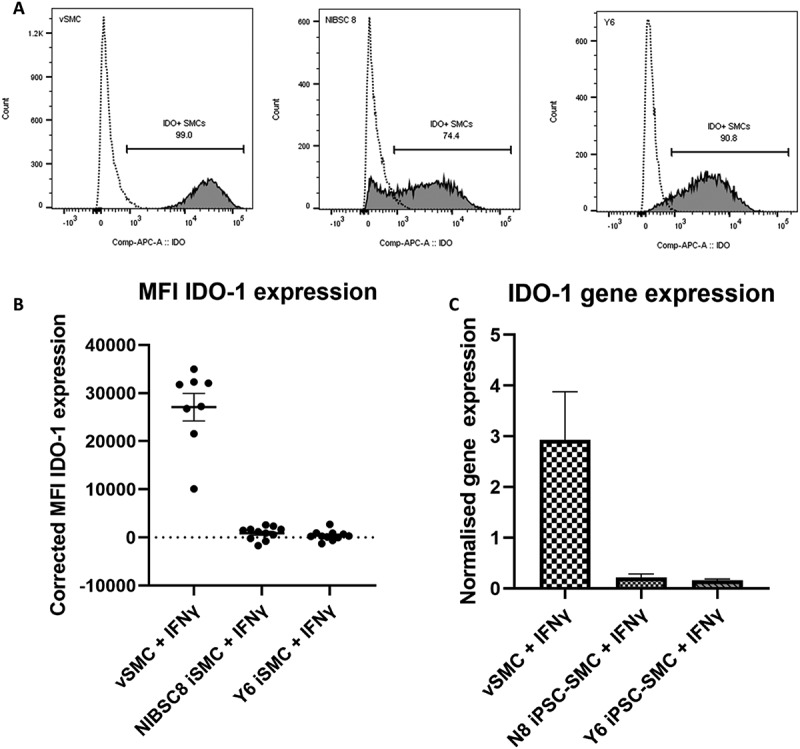
Indoleamine 2,3, dioxygenase (IDO-1) expression in induced pluripotent stem cell derived smooth muscle cells (iPSC-SMC) vs vascular smooth muscle cells (v-SMC). (A) Representative data of %IDO-1^+^ cells in primary v-SMC, and two iPSC-SMC lines; N8 and Y6 cell cultures (left to right). Dashed Line = Untreated. Grey = 72 hours of IFN-γ treatment, 50 ng/ml. (B) Corrected Mean fluorescence (MFI) (MFI treated – MFI untreated) of IDO-1 expression in IDO-1+ cells (*n* = 8 for v-SMC, *n* = 12 for Y6 iPSC-SMC and *n* = 11 for N8 iPSC-SMC, each from three individual assays). (C) Gene expression of IDO-1 in IFN-γ treated cells (50 ng/ml) after 72hrs. IDO-1 expression normalised to Glyceraldehyde-3-phosphate dehydrogenase (GAPDH) gene expression is shown expressed relative to the unstimulated cell control (*n* = 5 for iPSC-SMC lines, *n* = 2 for v-SMC line, from two independent experiments).

To establish whether iPSC-SMCs resemble their somatic counterpart regarding IDO-1 expression, we used a reductive assay system to monitor the influence of IFN-γ stimulation on IDO-1 expression. Cells were exposed to IFN-γ for 72hrs, after which IDO-1 expression was analyzed by flow cytometry (Figure 2 and Supplementary Figure S3). An additional iPSC-SMC cell line, Yale 6 (Y6), was included in these assays to determine if consistent levels of IDO-1 expression could be observed across different iPSC derived SMC lines. Flow cytometric analysis shows that neither the v-SMC nor the iPSC-SMC lines N8 or Y6 expressed IDO-1 without stimulation (mean IDO-1 expression levels across three separate assays were 2.3% in v-SMC, 3.3% in N8 iPSC-SMC, and 3.0% in Y6 iPSC-SMC). However, when exposed to IFN-γ for 72hrs, the mean fluorescence intensity (MFI) highlights differences in IDO-1 expression between the iPSC-SMC and v-SMC lines. The corrected MFI (MFI treated – MFI untreated) was used to quantify IDO-1 expression across the three individual assays (Figure 2(B)), and highlights that, while all cell types expressed IDO-1% of IDO^+^ cells increased to 91.4% in v-SMCs, 77.0% in N8 SMCs and 79.4% in Y6-SMCs, Figure 2(A)), levels in v-SMC > 10-fold higher in intensity than either iPSC-SMC (MFI: 1823 in N8-iPSC-SMC, 1655 in Y6-iPSC-SMC, and 23,297 in v-SMC), indicating that the iPSC-SMC lines were unable to induce IDO-1 to the same extent as their somatic cell counterpart. There was also some heterogeneity in IDO-1 expression indicated in NIBSC8-SMC (Figure 2(A)). The gene expression level of IDO-1 in IFN-γ stimulated v-SMC, N8 iPSC-SMC, and Y6 iPSC-SMC was also quantified by qPCR (Figure 2(C)). Reduced IDO-1 gene expression was also observed for the iPSC derived SMC lines in comparison with the primary v-SMC after IFN-γ stimulation (Figure 2(C)).

**Figure 3.**
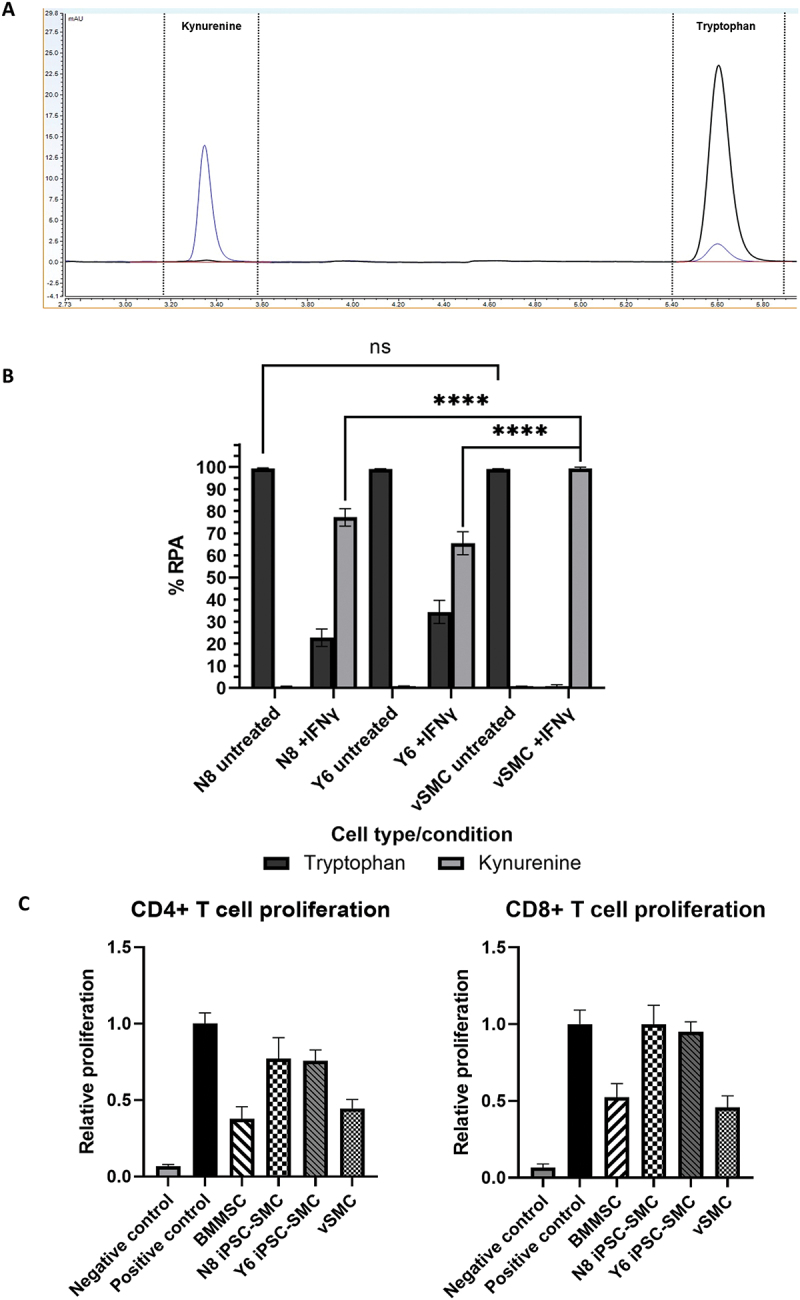
Induced pluripotent stem cell derived smooth muscle cells (iPSC-SMC) display reduced indoleamine 2,3 dioxygenase-1 IDO-1 activity and immunosuppressive capacity. (A) Representative chromatogram showing tryptophan (peak at ~5.65 mins) and kynurenine (peak at ~3.35 mins) levels in vascular smooth muscle cell (v-SMC) cultures with and without IFN-γ treatment. Black = not treated, Blue = iPSC-SMC culture after 72 hours incubation with IFN-γ (50 ng/mL). (B) Graph showing percentage relative peak area of Tryptophan and Kynurenine. (C) CD3^+^CD4^+^ and CD3^+^CD8^+^ T cell proliferation of PBMC co cultured with iPSC-SMC, bone marrow derived mesenchymal stromal cells (BMMSC) and v-SMC. Proliferation is measured as a percentage of Ki67^+^ expressing CD3+ cells and is expressed relative to the positive control (PBMC with CD3+/CD28+ bead stimulation). *n* = 5 from two independent experiments. Error bars represent ± SEM (*n* = 8 from three separate experiments). Multiple comparisons two-way ANOVA with Bonferroni post-test was performed. Asterisks denote statistical significance: *p ≤ 0.05, **p ≤ 0.01, ***p ≤ 0.001, ****p ≤ 0.0001.

To confirm IDO-1 functionality within the cells, media conditioned by v-SMC and iPSC-SMC were analyzed by HPLC after IFN-γ stimulation for 72hrs to determine levels of tryptophan and kynurenine (Figure 3). A representative chromatogram is shown in Figure 3(A) and the relative peak areas of tryptophan and kynurenine from each test sample are shown in Figure 3(B). When untreated, both v-SMC and iPSC-SMC supernatants contain high levels of tryptophan (99.2% and 98.7%, respectively) and low levels of kynurenine (<1.3%). However, after IFN-γ treatment of v-SMCs, tryptophan levels dropped to undetectable levels (0.0%), and kynurenine increased (100.0% RPA) indicating functional IDO-1 enzyme activity, metabolizing the tryptophan in these cells. Both iPSC-lines (N8 and Y6) also displayed increases in Kyn in the treated condition, with media containing a mean RPA for kynurenine of 82.9% in N8 cells and 56.0% in Y6; however, these levels were significantly lower (when assessed by two-way analysis of variance/ANOVA) suggesting reduced functional IDO-1 enzyme activity compared to somatic cells.

Finally, to determine if the reduced IDO-1 expression observed for the iPSC-SMC lines was reflected in a concomitant reduction in immunosuppressive function, these cells were co-cultured with anti-CD3/anti-CD28 stimulated PBMCs in a transwell format. A reduction in T cell proliferation (indicative of immunosuppression) was measured by the percentage of Ki-67 expressing cells (Figure 3(C)). As an immunosuppressive cell type control, bone marrow derived mesenchymal stromal cells (BMMSCs) were also included in the assay along with v-SMC. As expected, a considerable reduction in both CD4^+^ and CD8^+^ T cell proliferation was observed for BMMSC versus the stimulated PBMC control (relative CD4+ proliferation 0.38 ± 0.18, relative CD8+ proliferation 0.52 ± 0.20). The two iPSC-SMC lines showed some reduction in T cell proliferation, however, not to the same degree as the BMMSCs and behaved more similarly to the stimulated PBMC control (relative CD4+ proliferation 0.77 ± 0.31 and 0.76 ± 0.16; relative CD8+ proliferation 0.99 ± 0.28 and 0.95 ± 0.15 for N8 and Y6 iPSC-SMC respectively). In contrast, v-SMC did induce an immunosuppressive effect on the T cell populations similar to that of the BMMSC (relative CD4+ proliferation 0.45 ± 0.13; relative CD8+ proliferation 0.46 ± 0.17) supporting previous studies that show v-SMC are able to suppress the immune response [16,31]. As the assay was performed in transwell, without contamination from PBMC populations, the iPSC-SMC, v-SMC, and BMMSC were also analyzed for expression of the IDO-1 gene by qPCR (supplementary Fig S4). These data show reduced expression of IDO-1 in response to the activated PBMC in the assay in both iPSC-SMC populations compared with v-SMC and BMMSC.

**Figure 4.**
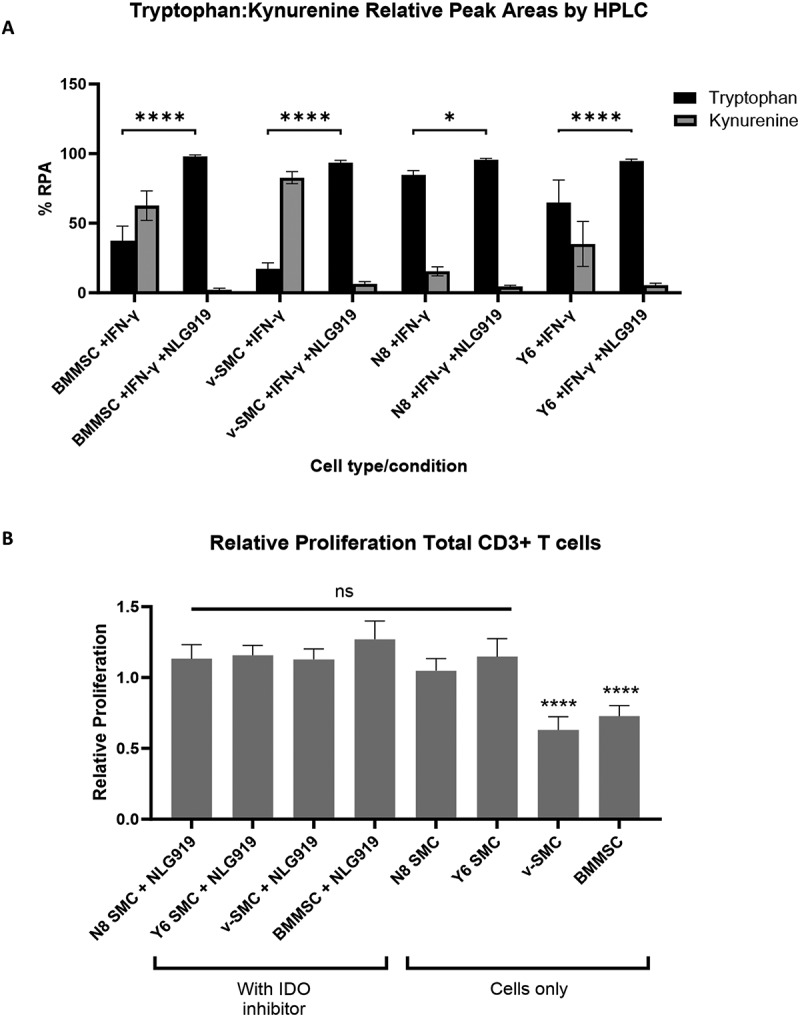
Indoleamine 2,3 dioxygenase-1 IDO-1 inhibition inhibits T cell suppressive ability of vascular smooth muscle cells (v-SMC). (A) Graph showing percentage relative peak area of Tryptophan and Kynurenine in IFN-γ stimulated SMC and bone marrow derived mesenchymal stromal cell (BMMSC) cultures with and without IDO inhibitor NLG919. *n* = 6 from three experiments (B) Graph showing relative CD3+ T cell proliferation of PBMC in a transwell co-culture with iPSC-SMC, BMMSC and v-SMC with and without IDO inhibitor treatment, NLG919. Proliferation is measured as a percentage of Ki67+ expressing CD3+ T cells and is expressed relative to the positive control (PBMC with Dynabead stimulation). Error bars represent ± SEM (*n* = 11 from four independent experiments). Statistical analysis was performed using a Mixed effects model with Dunnett’s multiple comparisons test. Asterisks denote statistical significance: ns = *p* > 0.05, *p ≤ 0.05, **p ≤ 0.01, ***p ≤ 0.001, ****p ≤ 0.0001.

To further investigate the role of IDO-1 in immunosuppression in these assays, an inhibitor of IDO-1, NLG919 [37,38] was included into cultures of iPSC-SMC, v-SMC, and BMMSC as a positive control, with and without additional IFN-γ stimulation to induce IDO-1 expression (Figure 4(A)). After a 72 hr incubation, supernatants were analyzed for levels of tryptophan and kynurenine by HPLC as described above. The relative peak areas (RPA, %RPA = (Peak Area of Analyte/Total Peak Area)*100) of tryptophan and kynurenine from each test sample are shown in Figure 4(A). Confirming the action of the IDO-1 inhibitor, NLG919, there is an inhibition of the conversion of tryptophan to kynurenine in all SMCs in all cultures treated with inhibitor (RPA for kynurenine in IFN-γ stimulated cultures with inhibitor of 97.8% in BMMSC, 93.6% in v-SMC, 95.5% in N8, and 94.5% in Y6 compared with stimulated cultures without inhibitor of 37.4% in BMMSC, 17.3% in v-SMC, 84.6% in NIBSC8-SMC and 64.9% in Y6-SMC). The inhibition of kynurenine conversion is significant for all lines, although to a lesser extent in the NIBSC8-SMC. Importantly, pharmacological inhibition of IDO erased the difference between the v-SMC and the iPSC-derived SMC lines.

After confirming the ability of NLG919 to inhibit IDO activity, this inhibitor was then incorporated into co-culture assays of the SMC lines with activated PBMC to assess the impact of IDO inhibition on the ability of the SMC lines to suppress T cell proliferation (Figure 4(B)). This was performed in a transwell format as before, to focus on the action of soluble factors of immunosuppression. Total CD3+ T cell proliferation is shown, expressed relative to proliferation in the positive (Dynabead stimulated) control. As anticipated, treatment of the BMMSC and v-SMCs with IDO inhibitor was able to reverse the suppression of CD3+ T cells observed with these immunomodulatory lines, with a relative proliferation of T cells in inhibitor treated co-cultures of v-SMC and BMMSC of 1.13 ± 0.07 and 1.27 ± 0.13, respectively, compared with 0.63 ± 0.09 and 0.73 ± 0.08 in co-cultures of v-SMC and BMMSC without inhibitor. Whilst the inhibition of T cell proliferation was significant between v-SMC and BMMSC and their inhibitor treated v-SMC counterparts, no difference in immunosuppression was found between either of the treated iPSC-SMC lines and their untreated counterparts, which gave a relative proliferation of CD3+ T cells of 1.05 ± 0.09 and 1.15 ± 0.13 in N8 and Y6-SMC untreated co-cultures, respectively, compared with 1.13 ± 0.10 and 1.15 ± 0.07 in N8 and Y6-SMC co-cultures with IDO inhibitor. There was no significant difference between inhibitor treated v-SMC and N8 or Y6-SMC, indicating that treatment of v-SMC co-cultures with IDO inhibitor reduced their ability to suppress proliferation of CD3+ T cells to a level that was similar to that of the iPSC-SMC lines. The reduced immunosuppressive capacity of the two iPSC-SMC lines shown here suggests that iPSC-SMC lack the immunosuppressive capability found in their somatic counterpart, and that this is likely due to a reduced ability to express IDO-1 in response to an inflammatory environment.

## 4. Discussion

There are now convincing data demonstrating that v-SMCs exhibit a non-immunogenic phenotype [14,16,35,39,40]. The immune profile of iPSC-derived SMCs, however, is disputed, with conflicting evidence from recent studies suggesting various levels of immunogenicity [17–20]. This observed immune response, and the ways in which it differs from naturally differentiated cells, is particularly relevant when considering potential applications in regenerative medicine. If TEVGs are to be available “off the shelf,” it is likely that the use of allogeneic cells will be key. For example, one route to clinic may be the maintenance of a bank of iPSC-derived cells with different MHC subtypes. For this reason, it is important to fully characterize and understand the immunogenic potential of the iPSCs derived cells.

In this study, we sought to further investigate the observed immunogenicity of iPSC-SMCs in comparison with their naturally differentiated counterpart, v-SMC [21]. The expression of a panel of immune-related antigens involved in the activation of T effector memory (T_EM_) cells, considered to have an important role in allograft rejection [15,30], was compared in the two cell types. Among the antigens tested, HLA Class I was investigated as a primary mediator of immunogenicity. Data revealed a high basal expression of HLA Class I in all test cell types (iPSC-SMCs, v-SMCs, and ECs). Interestingly, expression of OX40-L, which has been shown to contribute to immunogenicity of ECs [16,27], was negligible in the iPSC-SMCs. The iPSC-SMC line also showed reduced expression of co-stimulatory molecules; CD86, ICOS-L, and LFA-3 compared to their v-SMC counterpart. CD86 expression is strongly linked to T cell activation via the direct pathway of allorecognition; requiring the formation of complexes between CD28 on CD4+/CD8+ T cells and CD86 on the donor antigen presenting cell (APC) [41,42], alongside HLA-antigen complex and T cell receptor binding. It has also been observed that, in humans, ICOS-L can serve as an alternative ligand for CD28 and CTLA-4 [43]. The reduced expression of co-stimulatory molecules on the cell surface may therefore suggest that the immunogenic phenotype of iPSC-SMCs is mediated by the indirect, or semi-direct antigen presentation pathway.

Endothelial cells were used in our assays as a positive control due to their known pro-immunogenic activity, stimulating T_EM_ proliferation through increased expression of co-stimulatory molecules (Figure 1(C)). Although not to the same extent, iPSC-SMC also stimulated proliferation of both CD4+CCR7- and CD8+CCR7- T_EM_ in whole PBMC co-culture assays, in contrast to the negligible proliferation observed in response to v-SMCs included in the same assay. However, since no similar increase in co-stimulatory molecules was seen in the iPSC line, the T_EM_ proliferation observed with these cells (Figure 1(B)) is likely through another mechanism. Also of interest, neither iPSC-SMC nor v-SMC elicited a response to CD3+ T cells only, indicating a more complex interaction with other cell types present in PBMC is required to activate T cell proliferation. It will be of importance in future studies to examine the role of different cell types using a range of additional assays to determine immunogenicity and immunomodulatory function [41], in addition to the inclusion of a wider screen of co-stimulatory molecules that may be involved in the immunogenicity observed.

SMCs have previously been shown to reduce an inflammatory response through IDO-1 mediated immunomodulatory activity [14,16]. We therefore sought to examine IDO-1 expression in iPSC-SMC and v-SMC when stimulated with the pro-inflammatory cytokine IFN-γ to determine if the iPSC-SMCs had reduced immunomodulatory function. Both iPSC-SMC and v-SMC showed an increase in IDO-1+ cells upon stimulation with IFN-γ. Critically, expression levels in v-SMC were approximately 10-fold higher than that of two distinct iPSC-SMC lines.

Confirmatory data from qPCR showed a similar reduced IDO-1 expression in the iPSC-SMC lines compared with v-SMCs, indicating that reduced IDO-1 results from a lack of gene induction/expression rather than any post-translational modifications. As lower levels of IDO-1 expression presumably correlate with a reduced tryptophan catabolism, we investigated this by assessing the functional activity of the IDO-1 expressed by iPSC-SMCs using HPLC to analyze cell supernatants for tryptophan (Trp) and kynurenine (Kyn). In the presence of IFN-γ, Kyn increased in v-SMCs with no detectable remaining Trp, indicating complete conversion of all tryptophan in the supernatants of the v-SMC co-cultures. However, in IFN-γ treated iPSC-SMCs, although Kyn levels increased, these were not to the levels seen in v-SMCs, and Trp levels remained detectable, indicating that lower levels of IDO-1 expressed in the iPSC-SMCs were indeed leading to reduced tryptophan catabolism. To confirm their reduced immunomodulatory activity, iPSC-SMCs were less able to suppress T cell proliferation when co-cultured with stimulated lymphocytes compared with v-SMC. Addition of an inhibitor of IDO-1, NLG919, was able to abrogate the immunosuppression of v-SMC and render their activity in the co-culture similar to that of the iPSC-SMC, indicating the expression of IDO-1 is of particular importance to the v-SMC immune modulatory phenotype exhibited [14,16]

In addition to provoking an immune response through antigen expression, the importance of immune modulation of iPSC derived cell types now must be considered [21,44–46]. Aside from the physiological role that the induction of IDO-1 in v-SMC plays in contributing to medial immunoprivilege, the induction of IDO-1 may also be of particular advantage when considering the induction of a tolerogenic microenvironment to promote graft acceptance of a cellular therapy. It has been shown previously that IDO-1 expression from other iPSC derivatives such as hepatocytes [44] and mesangioblasts [45], is responsible for suppression of T cell proliferation in *in vitro* assays, and as described above, IDO-1 expression is a key component of the immunosuppressive capability of vessel derived SMC. A reduction in IDO-1 expression may indicate an overall reduced ability of the iPSC-SMCs to respond to inflammatory stimuli, which in some instances may be useful, for example, in graft acceptance, but in others may hinder the cells’ own protection capabilities. The therapeutic relevance of the different immune phenotypes observed between iPSC-SMC and v-SMC in this study will need to be confirmed in further *in vitro*, and preferably *in vivo*, studies. However, from our data we can infer that the reduced expression of IDO-1 in iPSC-SMC may be one of the key mechanisms involved in the different immune phenotypes observed in this study. These phenotypes may be influenced by cell-specific factors such as epigenetic memory, genetic mutations introduced from the original donor, reprogramming methods or culture conditions. The level of cell maturity may also play a role, and indeed both cardiomyocytes and pancreatic islet cells have shown that a lack of structural maturity results in reduced function [9,19,41,47–52]. Furthermore, it has been observed in both cell types, that application of an optimized maturation protocol improved cell-specific function [47–51]. Maturation may be of particular importance given that somatic cells acquire maturity during a longer developmental period in conjunction with their immunological surroundings in an *in vivo* niche [41].

Interestingly, the IDO-1 expression level was similarly reduced in two different iPSC- SMC lines, which have been differentiated using different methods ([21,24]). Expanding this study to incorporate additional iPSC lines (sources/reprogramming methods) and differentiation protocols will be useful in determining the role of these processes on the recapitulation of the IDO functional pathway. Furthermore, investigation into the maturation of iPSC-SMCs may give further insights into the potential differences that exist between mature v-SMC and iPSC derivatives. These additional studies will also be helpful in understanding the root cause of the reduced IDO-1 expression and function that has been observed here, for example, if it results from an impaired ability to respond to the inflammatory environment through reduced receptor expression, or if epigenetic differences in the IDO-1 promoter may play a part.

With the likelihood that cell therapies will use allogeneic sources of material, there are a number of avenues being explored to circumvent the immune response. Standardized iPSC lines from HLA banks and genetically modified iPSC lines, engineered to inactivate HLA class I and II to render them hypoimmunogenic, may serve as a route around immunogenicity of cells that result from overexpression of immune-related antigens, particularly in an allogeneic transplant setting [53–56]. However, although techniques and efficacy have improved in genetic manipulation [57], there is still a gross risk of off-target effects. This is accompanied by the acknowledgment that an immune-eluding cell line may well pose a risk of cancer development to the transplant recipient [41].

### 4.1. Conclusion

With the increasing focus on induced pluripotent stem cells (iPSCs) for use in regenerative therapies,it is clear that while thorough characterization of the terminally differentiated cell phenotype is essential, effective clinical application of iPSC-based therapies will also require an improved understanding of immunological differences between iPSC derivatives and their somatic counterparts.

iPSC-derived smooth muscle cells (iPSC-SMCs) have great potential in the application to tissue engineered vascular grafts/blood vessels (TEVGs/TEBVs), for the treatment of vascular diseases.v-SMCs are immunomodulatory, and therefore mirroring this non-immunogenic profilewould be essential in the application of any iPSC-SMC to this therapy. The data presented here highlights differences in the immune profile of iPSC-SMC and v-SMC. Further investigation of IDO-1 activity in iPSC-SMCs, particularly within physiologically relevant *in vivo* environments, will be an important next step in robust characterization, prior to reaching the clinic.

More broadly, this work supports the need to incorporate immune functional assessment alongside conventional phenotypic analysis in the development of iPSC-derived therapies.

## Supplementary Material

Supplemental Material

Supplementary figure legends and supplementary methods.docx

## Article highlights

- Smooth Muscle Cells (SMCs) derived from induced Pluripotent Stem Cells (iPSCs) have significant potential in cellular therapy for many cardiovascular diseases, but there is currently limited understanding of iPSC-SMC immunological profiles, required to advance their clinical application.
- This article describes the investigation of the functional immunogenicity of iPSC-derived SMCs compared to naturally derived vascular SMC (v-SMC), with the following conclusions:
- In contrast to v-SMC, iPSC-SMC trigger T effector memory (T_EM_) proliferation.
- This immunogenicity is not due to over expression of T_EM_ activation-related antigens, as these are shown to be comparable between the two cell types.
- Indoleamine 2,3 dioxygenase (IDO-1) expression and activity was investigated, as it has been shown that arterial v-SMC can establish immunoprivilege through IDO-1. Significantly reduced IDO-1 expression and functionality was shown in two independent iPSC-SMC lines compared to the v-SMC.
- In contrast to v-SMC, neither iPSC-SMC line could modulate the immune response in a co-culture with CD3/CD28 activated Peripheral Blood Mononuclear Cells (PBMC)
- Overall, our data indicates that the difference in immunogenicity of iPSC-SMC compared to their vascular counterpart is due to an impaired ability of iPSC-SMC to modulate the immune response through IDO-1 activity.

## Disclsoure statement

The authors have no relevant affiliations or financial involvement with any organization or entity with a financial interest in or financial conflict with the subject matter or materials discussed in the manuscript. This includes employment, consultancies, honoraria, stock ownership or options, expert testimony, grants or patents received or pending, or royalties.

No writing assistance was utilized in the production of this manuscript.

## Data and material availability Statement

The data that support the findings of this study are available from the corresponding author, Katherine Partridge, upon reasonable request.

There are restrictions to the availability of iPSC-derived SMC line N8 due to the lack of an external centralized repository for their distribution and our need to maintain stocks. We will consider reasonable requests for iPSC-derived SMC line N8 with potential compensation by requestor for its processing and shipping. We may require a completed Material Transfer Agreement if there is potential for commercial application. The untransformed human aortic smooth muscle cells and Y6 iPSC-derived SMC were donated to the study gifts, we are therefore unable to authorize the distribution of these materials. Further information and requests for resources and reagents should be directed to the corresponding author.
