# Supplementary material for: Immunogenicity of induced pluripotent stem cell-derived smooth muscle cells results from a reduction in the expression of indoleamine 2,3 dioxygenase (IDO-1)
Source: Regen Med. 2026 Feb 25;21(1):9–20. doi: 10.1080/17460751.2026.2631599 (PMC13011584; doi:10.1080/17460751.2026.2631599)
Supplement: Supplemental Material [file IRME_A_2631599_SM2756.zip › IRME_A_2631599 Suppl figures and tables/Supplementary_Tables_S3B_S3C.docx]

| Cell line/treatment | MFI IDO expression | | | | | | | | | | | | Mean | SEM | n |
| --- | --- | --- | --- | --- | --- | --- | --- | --- | --- | --- | --- | --- | --- | --- | --- |
| vSMC unstimulated | 808 | 4556 |  | 1246 | 1501 | 1538 | 3138 | 1320 | 2072 |  |  |  | 2022 | 1239 | 8 |
| vSMC + IFNγ | 32879 | 31793 |  | 28005 | 33276 | 33840 | 13235 | 36284 | 23650 |  |  |  | 29120 | 7518 | 8 |
| NIBSC8 iSMC unstimulated | 1758 | 5416 | 4290 | 1529 | 2634 | 3221 | 807 | 837 | 844 | 1541 | 1381 |  | 2205 | 1527 | 11 |
| NIBSC8 iSMC + IFNγ | 4108 | 3739 | 3518 | 4116 | 4108 | 3046 | 2490 | 2010 | 2514 | 2115 | 2262 |  | 3093 | 850 | 11 |
| Y6 iSMC unstimulated | 1582 | 2052 | 3089 | 2599 | 3058 | 1049 | 1348 | 1518 | 2670 | 1268 | 1186 | 1254 | 1889 | 766 | 12 |
| Y6 iSMC + IFNγ | 2505 | 2341 | 2485 | 3497 | 3746 | 3769 | 1453 | 1509 | 1384 | 1492 | 1501 |  | 2335 | 960 | 11 |
|  |  |  |  |  |  |  |  |  |  |  |  |  |  |  |  |
| Cell line/treatment | % IDO expression | | | | | | | | | | | | Mean | SEM | n |
| vSMC unstimulated | 0.3 | 1.8 |  | 0.2 | 0.1 | 0.1 | 2.0 | 5.8 | 8.4 |  |  |  | 2.3 | 3.1 | 8 |
| vSMC + IFNγ | 98.6 | 98.4 |  | 99.1 | 99.3 | 99.4 | 45.9 | 72.6 | 72.5 |  |  |  | 85.7 | 20.0 | 8 |
| NIBSC8 iSMC unstimulated | 1.2 | 0.8 | 8.6 | 0.0 | 0.1 | 0.0 | 1.1 | 0.9 | 2.2 | 12.9 | 8.5 |  | 3.3 | 4.5 | 11 |
| NIBSC8 iSMC + IFNγ | 66.5 | 76.7 | 74.1 | 70.0 | 74.1 | 72.1 | 86.2 | 79.8 | 84.8 | 80.1 | 82.4 |  | 77.0 | 6.3 | 11 |
| Y6 iSMC unstimulated | 0.5 | 0.1 | 0.8 | 0.1 | 0.1 | 0.0 | 6.0 | 9.2 | 13.0 | 2.8 | 1.2 | 1.8 | 3.0 | 4.2 | 12 |
| Y6 iSMC + IFNγ | 90.7 | 90.1 | 87.7 | 91.0 | 93.0 | 93.2 | 67.0 | 69.5 | 56.9 | 63.9 | 70.8 |  | 79.4 | 13.8 | 11 |
|  |  |  |  |  |  |  |  |  |  |  |  |  |  |  |  |

**Figure S3: IDO-1 expression in iPSC-SMC and vSMC, related to Fig 2**

1. Representative histograms showing expression of IDO-1 in unstimulated and 50ng/ml IFN-γ treated cells after 72hrs. Isotype controls for each line is shown in top panel. (B) Table showing % IDO expression in each iPSC-SMC line with mean, SEM. (C) Table showing mean fluorescence intensity (uncorrected) in each iPSC-SMC and vSMC line with mean, SEM.
