# Supplementary figures and images for: Immunogenicity of induced pluripotent stem cell-derived smooth muscle cells results from a reduction in the expression of indoleamine 2,3 dioxygenase (IDO-1)
Source: Regen Med. 2026 Feb 25;21(1):9–20. doi: 10.1080/17460751.2026.2631599 (PMC13011584; doi:10.1080/17460751.2026.2631599)

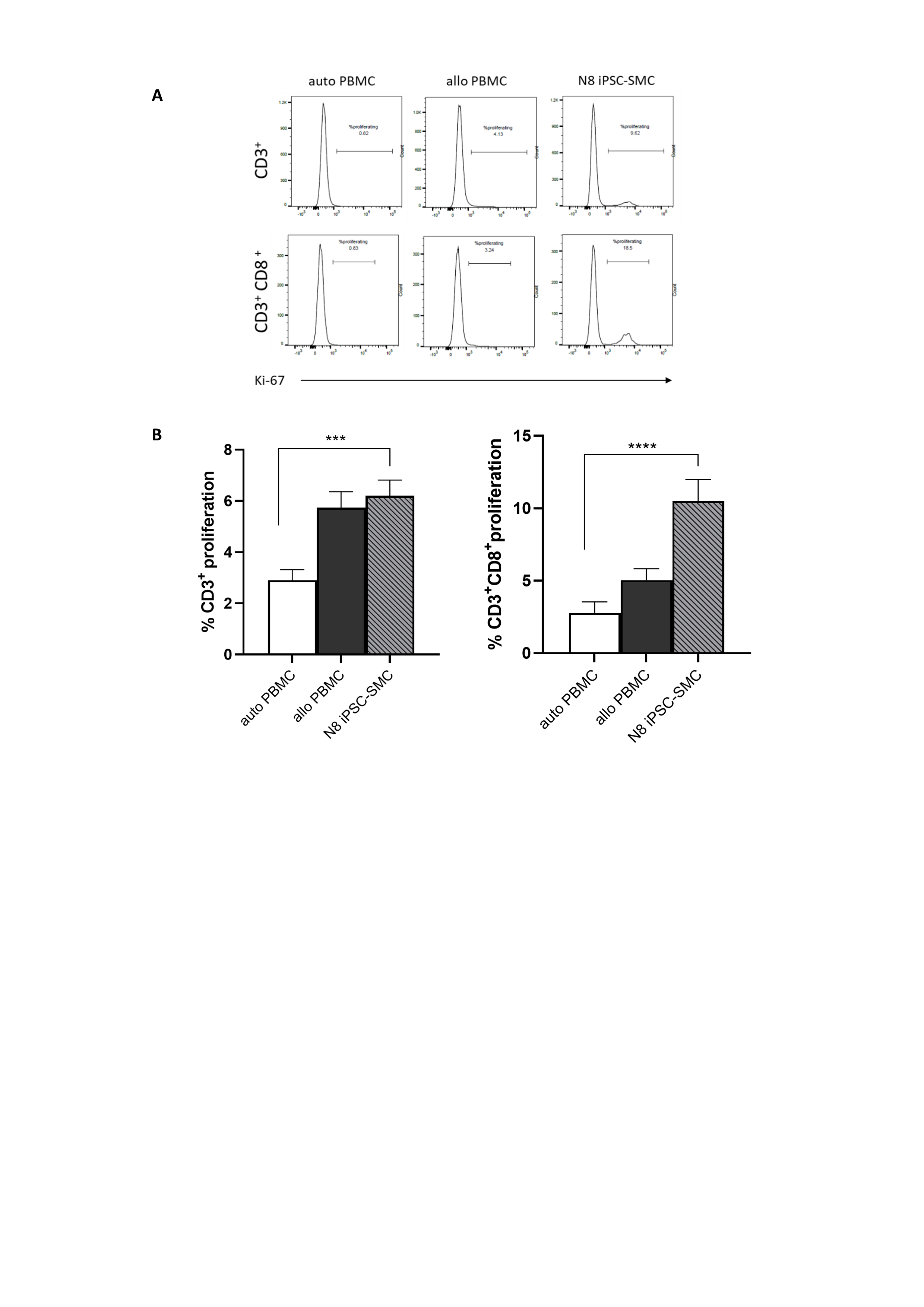

Supplement: Supplemental Material [file IRME_A_2631599_SM2756.zip › IRME_A_2631599 Suppl figures and tables/FigS1_high res.png]

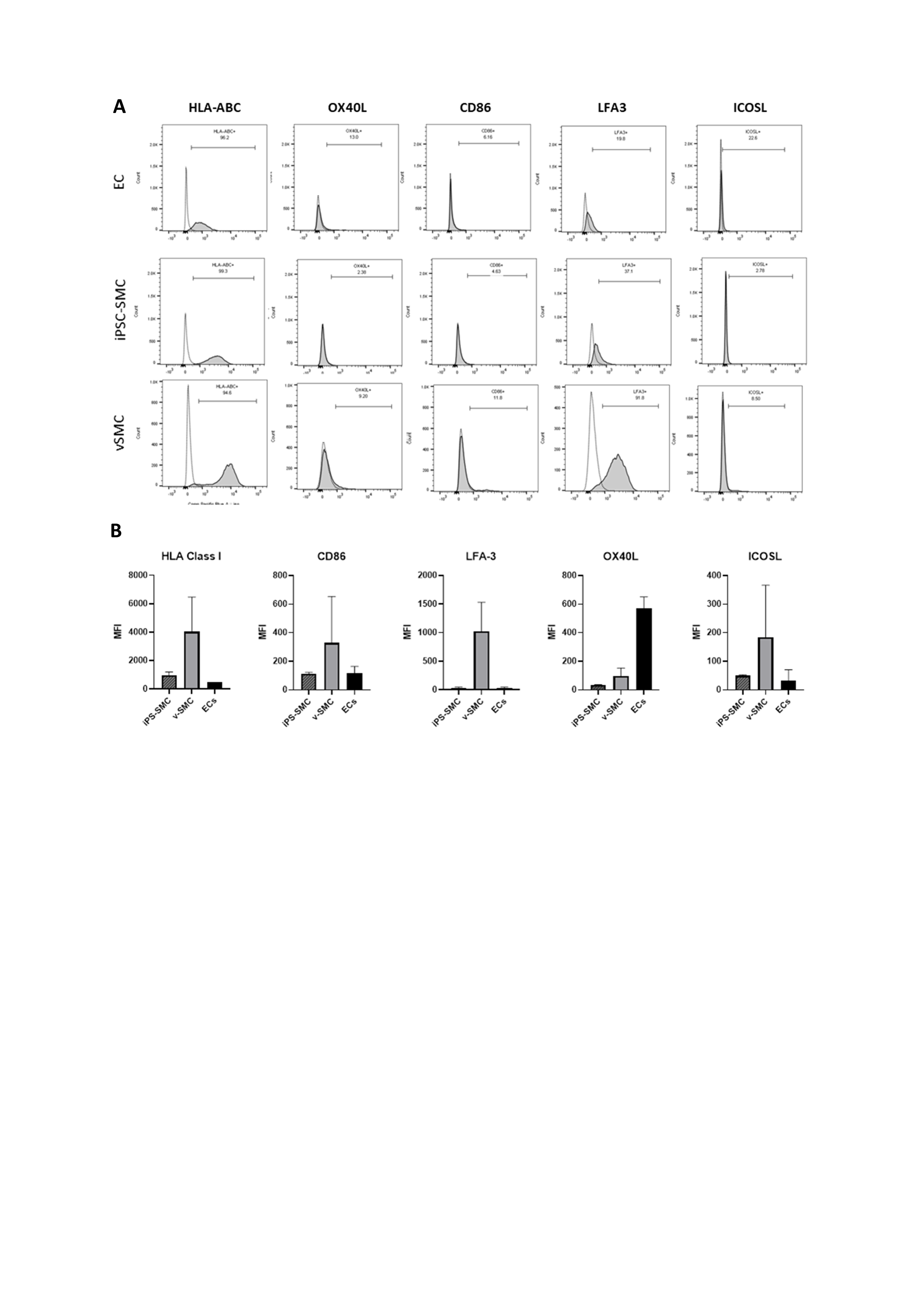

Supplement: Supplemental Material [file IRME_A_2631599_SM2756.zip › IRME_A_2631599 Suppl figures and tables/FigS2_high res.png]

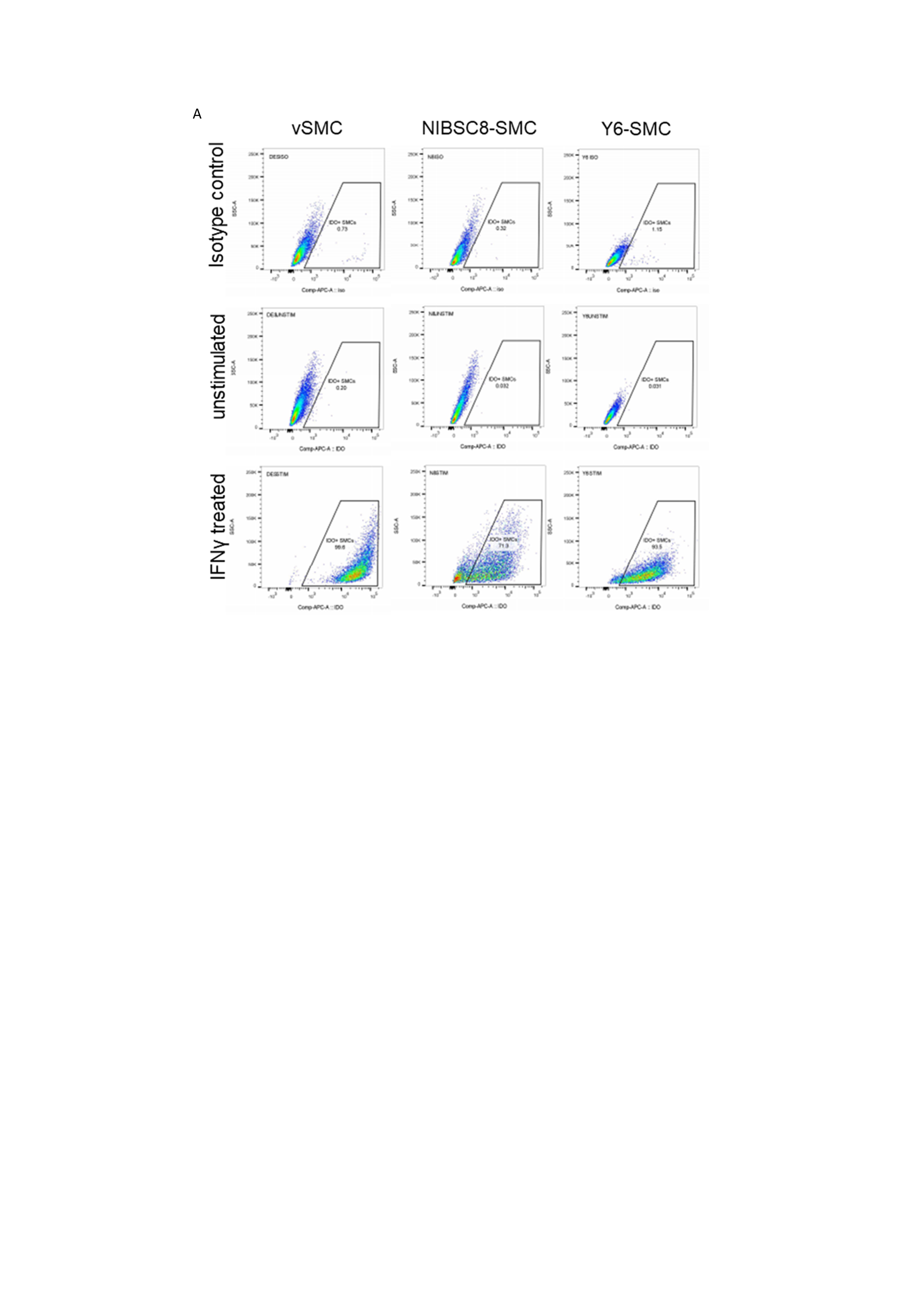

Supplement: Supplemental Material [file IRME_A_2631599_SM2756.zip › IRME_A_2631599 Suppl figures and tables/FigS3A_high res.png]

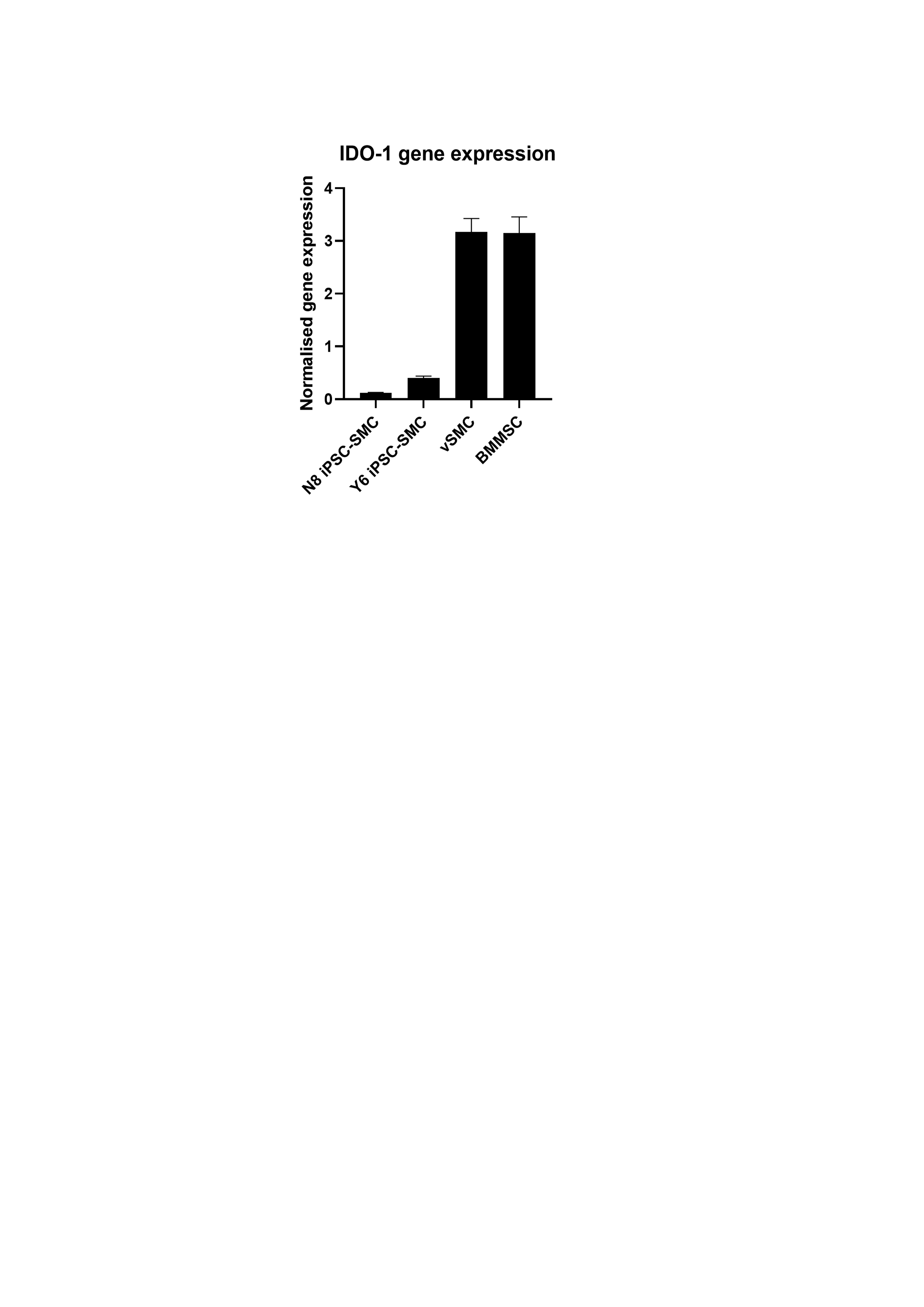

Supplement: Supplemental Material [file IRME_A_2631599_SM2756.zip › IRME_A_2631599 Suppl figures and tables/FigS4_high res.png]
