# Supplementary material for: Immunogenicity of induced pluripotent stem cell-derived smooth muscle cells results from a reduction in the expression of indoleamine 2,3 dioxygenase (IDO-1)
Source: Regen Med. 2026 Feb 25;21(1):9–20. doi: 10.1080/17460751.2026.2631599 (PMC13011584; doi:10.1080/17460751.2026.2631599)
Supplement: Supplementary figure legends and supplementary methods.docx [file IRME_A_2631599_SM3888.docx]

**11. Supplementary figure legends**

**Figure S1. iPSC derived SMC stimulate lymphocyte proliferation.**

1. Representative histograms show CD3+ and CD3+CD8+ T cell proliferation of responder cells after 5 days in co culture with either autologous (auto) PBMC, allogeneic (allo) PBMC or iPSC derived SMC (N8 iPSC-SMC). (B) Graphs showing percentage proliferation (% Ki-67+ cells) of total CD3+ and CD3+CD8+ T cell populations in responder cells in response to the stimulator cells, autologous (auto) PBMC or allogeneic (allo) PBMC after 5 days in co culture. Error bars represent ± SEM (n=11 biological replicates from three separate experiments). Ordinary one-way ANOVA with Bonferroni post-test was performed. Asterisks denote statistical significance: * p ≤ 0.05, ** p≤ 0.01, *** p ≤ 0.001, **** p ≤ 0.0001.

**Figure S2: Co-stimulatory marker expression in in ECs, v-SMC and iPSC-SMC, related to Figure 1C**

1. Representative histograms show expression of HLA class I and costimulatory markers CD86, Ox40-L, LFA3 and ICOS-L (shaded histogram) in ECs, iPSC-SMC and vSMC cells. Corresponding % expression is shown above gate. Relevant isotype control is shown in dashed line. (B) Graphs showing corrected Mean Fluorescence Intensity (MFI) of expression of immune related antigens; HLA class I, CD86, OX40-L, and ICOS-L in N8 iPSC-SMC, v-SMC and EC populations assessed via flow cytometry. Error bars represent +/- SEM (n= 2 from independent experiments).

**Figure S3: IDO-1 expression in iPSC-SMC and vSMC, related to Fig 2**

1. Representative histograms showing expression of IDO-1 in unstimulated and 50ng/ml IFN-γ treated cells after 72hrs. Isotype controls for each line is shown in top panel. (B) Table showing % IDO expression in each iPSC-SMC line with mean, SEM. (C) Table showing mean fluorescence intensity (uncorrected) in each iPSC-SMC and vSMC line with mean, SEM.

**Figure S4. IDO-1 expression in SMC populations exposed to activated PBMC, related to Figure 4.**

1. Graph showing expression of IDO-1, normalised to GAPDH, in SMC populations in response to CD3/CD28 Dynabead activated PBMC after 5 days in co culture. In a transwell set up. SMC populations were plated at the bottom of a 24 well plate, with PBMC and Dynabeads in the transwell upper compartment. Error bars represent ± SEM (n=3biological replicates).

**Supplemental Experimental Procedures, related to Experimental Procedures (Section 4).**

Cell culture – maintenance and passage

N8 iPSC Certificate of Analysis, including karyotyping and analysis of pluripotency, can be found on the hPSC registry at: https://hpscreg.eu/cell-line/USCBi001-A. Y6 iPSC were derived and characterised, including karyotyping and analysis of pluripotency, as described in [41].

N8 iPSC-SMC were maintained in SMC expansion media (Dulbecco's Modified Eagle Medium, DMEM, Gibco™, Loughborough, UK) containing 5 ng/mL FGFβ, 5% v/v foetal calf serum (FCS) and 2 mM L-Glutamine (Sigma-Aldrich, Dorset, UK).

Y6 iPSC-SMC were cultured on 0.1% gelatin-coated plates, in DMEM containing 5% v/v FCS, 2 mM L-Glutamine, 0.1 mM β-mercaptoethanol (Gibco™), and 1% v/v non-essential amino acids (Gibco™). Y6 iPSC-SMC and N8 iPSC-SMC were used between passage 5-10. Vessel-derived SMCs were obtained from descending or ascending tract and designated v-SMC. v-SMC were cultured in Medium-199 (Sigma-Aldrich), 10% v/v heat-inactivated FCS, 2 mM L-glutamine, 100 U/mL penicillin and 100 mg/mL streptomycin (Sigma-Aldrich). Cells were used between passage 3-8. HUVECs were cultured on 0.1% gelatin-coated plates in complete EGM-2 MV media (Lonza Bioscience, Switzerland). Cells were used between passage 3-5.

BM-MSCs were maintained in DMEM, 10% v/v FCS, 2 mM L-glutamine, 100 U/mL penicillin and 100 mg/mL streptomycin.

PBMC isolated by Histopaque®-1077 from whole blood were stored over liquid nitrogen for use in immune-suppression or immune-modulation assays. PBMC were cultured in R10 (RPMI 1640 (Sigma-Aldrich), 10% v/v FCS, 2 mM L-glutamine, 50 U/mL penicillin/streptomycin).

One-way MLR and immunosuppression assays

For the one-way MLR to assess immunogenicity, co-cultures of 3x105 responder PBMC or isolated T cells and 1.5x105 SMC stimulator cells were maintained in 200 μL of complete R10 in 96-well plates at 37⁰C in 5% CO2 for 5 or 7 days. Where used, T cells were isolated using Dynabeads FlowComp Human CD3 (ThermoFisher, Loughborough, UK) according to the manufacturer’s instructions. Proliferation of responder cells was assessed with either carboxyfluorescein succinimidyl ester (CFSE, ThermoFisher/Invitrogen) labelling prior to the start of co-culture, or by staining for the intracellular marker, Ki-67, at the end of the co-culture using BD Cytofix/Cytoperm (BD Biosciences). As a positive control for T cell activation, PBMCs were stimulated with Dynabeads® Human T-Activator CD3/CD28 (Gibco™) according to the manufacturer’s instructions. To prevent the detection of proliferation by stimulator SMCs, their proliferative capacity was disabled by UV irradiation. Harvested PBMC were stained with the fixable viability dye, eFluor660 (FVD, eFluor 660, Invitrogen) and the surface T cell markers CD3, CD4, CD8 and CCR7 (BD Biosciences). Cells were fixed with 4% PFA in PBS and analysed using BD FACS Canto II. Data was analysed using FlowJo 7.6.5.

For immune suppression assays, N8 iPSC-SMC, Y6 iPSC-SMC and v-SMC, in parallel with BM-MSC, were incorporated into the one-way MLR as described above, with some modifications. Cells were seeded at a density of 2.5x105 cells/well in a 24-well plate and incubated overnight in cell-specific growth media. PBMC from 4 donors were combined and added to the BM-MSC or

SMC at 4x105 PBMC in a transwell compartment. Additional activation stimulus was provided with the inclusion of Dynabeads as described above. Co-cultures were incubated at 37⁰C, 5% CO2 for 5 days, after which PBMC and Dynabeads were removed and PBMC stained with T cell markers CD3, CD4 and CD8. Proliferation was assessed on BD FACS Canto II by Ki-67+ intracellular staining at the time of analysis. Data was analysed using Flowjo 10.8.1.

Indoleamine-2,3 dioxygenase-1 inhibition assays

To assess whether the reduced immunosuppressive activity in N8 and Y6 iPSC-SMC cells is due to limited IDO-1 activity in these cell lines relative to native v-SMCs, an assay was designed where we inhibited the IDO-1 activity in v-SMCs through the application of 6μM NLG919 (Navoximod, Abcam, Cambridge, UK), a selective IDO-1 inhibitor. NLG919 was diluted in assay medium and included in SMC cultures with IFNγ stimulation for analysis by HPLC, and in co-cultures of SMCs with PBMC to assess the affect on immunosuppression.

qPCR

Total RNA was isolated from cell lysates using the RNeasy Mini Kit (QIAGEN, London, UK) according to manufacturer’s instructions. First-strand cDNA synthesis from RNA was performed using the M-MLV Reverse Transcriptase system (Promega, Southampton, UK), and the cDNA product was subject to qPCR using 2 x qPCRBIO SyGreen Blue Mix Lo-ROX (PCRBiosystem, London, UK) and 400nM each pre-designed primers (Integrated DNA technologies, Belgium) Primers used were; IDO For 5’ AGAGTCAAATCCCTCAGTCC 3’; IDO Rev 5’ AAATCAGTGCCTCCAGTTCC 3’. Target gene expression was normalised to the expression of GAPDH (For 5’-ACG AAT TTG GCT ACA GCA ACA GGG-3’, Rev 5’-TCT ACA TGG CAA CTG TGA GGA GG-3’). qPCR was performed on the Rotor-Gene Q Thermocycler, and data analysed using Rotor-Gene Q System (QIAGEN, London, UK), 2.1.0

Reverse-Phase High Performance Liquid Chromatography (RP-HPLC)

Kynurenine and tryptophan content in supernatants from cell cultures was measured by HPLC as a readout of IDO-1 enzyme activity. N8 and Y6 iPSC-SMCs and v-SMCs were seeded into a 6-well plate and incubated with 50 ng/mL IFN-γ for 48hrs at 37⁰C, 5% CO2. After incubation, 200 μL supernatant was removed from culture, treated with 40 μL 30% v/v Trichloracetic acid (Sigma-Aldrich,) and further incubated at room temperature for 15 minutes prior to centrifugation (1500 rpm, 4⁰C, 5 min). Samples were filtered using Amicon Ultra-1- membrane spin column (Merck, Dorset, UK) and centrifuged at 13000 rpm, 4⁰C, 6 min. Filtered samples were loaded in to a Thermo Scientific UltiMate 3000 System, with an Agilent Zorbax SB-C18 column, 4.6x150 mm, 3.5 μm (Agilent, Oxford, UK), controlled by, and analysed on Chromeleon 7 Software. Mobile phase: 92.5% 10 mM Ammonium Formate pH 4.0 (Honeywell, Fisher Scientific, UK), and 7.5% Acetonitrile (VWR, Leicestershire, UK). Flow rate: 1 mL/min. 16 μL injection volume. Autosampler: 4⁰C, MWD UV: 280 and 360 nm. L-Trp and L-Kyn (Sigma-Aldrich) were diluted to form a standard curve for both products to enable peak identification.
